# Anticancer drug development against ribosome synthesis and the nucleolus

**DOI:** 10.1042/BST20253011

**Published:** 2025-08-04

**Authors:** Andrew Loiacono, Sui Huang

**Affiliations:** Department of Cell and Developmental Biology, Northwestern University Feinberg School of Medicine, Chicago, U.S.A.

**Keywords:** cancer, drug development, nucleolus, nucleus, ribosome biogenesis

## Abstract

Nucleoli, the most prominent nuclear organelle, form around ribosomal DNA (rDNA) clusters at the p-arms of the five acrocentric chromosomes. Nucleoli are centers of ribosome synthesis, a vital activity in cell proliferation and organism viability. Ribosome biogenesis is a complex process involving the activity of all three RNA polymerases and numerous cellular factors. This energy-consuming process is, therefore, highly regulated, with the transcription of rDNA being the rate-limiting step. Given that uncontrolled cell proliferation is a hallmark of cancer, enhanced ribosome biogenesis plays a crucial role in sustaining tumor growth. In addition, nucleoli are multi-functional organelles, participating in genome organization, cell cycle, stress sensing, macromolecular trafficking, and the sequestration of cellular factors—functions that are also significantly altered in cancerous conditions. This review focuses on summarizing the role of nucleoli in carcinogenesis and anticancer therapeutics that target nucleoli and ribosome synthesis.

## Ribosome synthesis and nucleoli

Since the discovery of nucleoli during the preliminary stages of microscopy development, knowledge about nucleolar structure and function has substantially expanded [1–6]. Nucleoli serve as the primary site of ribosome synthesis, facilitating and regulating this multi-step process [7]. Ribosome biogenesis involves transcription by RNA polymerase I (Pol I) using ribosomal DNA (rDNA) as the template to produce pre-rRNAs that are processed and subsequently assembled with corresponding large and small subunit ribosomal proteins to form 40S and 60S pre-ribosomal particles. These particles are then exported to the cytoplasm, where they undergo further maturation processes before becoming fully functional ribosomes [6,8–10]. Extensive studies at the cellular and molecular levels have revealed the basic mechanics that govern each step of ribosome synthesis, from the transcription of rDNA to the maturation of pre-ribosome particles in the cytoplasm. The transcription of rDNA within nucleoli yields polycistronic pre-rRNAs composed of 18S, 5.8S, and 28S rRNAs embedded in the 5′ and 3′ ETS and ITS1 sequences. The processing of the pre-rRNA involves complex, multi-step mechanics that cleave and modify the RNA to release each of the subunits [11,12]. The released rRNA subunits are then assembled with ribosomal proteins into the 40S and 60S pre-ribosomes, which are exported out of the nucleoli and nuclei to the cytoplasm for further maturation prior to becoming functional ribosomes. This multifaceted process encompasses the transcription by all three RNA polymerases along with many other cellular factors, whose functions collectively consume a substantial portion of cellular energy—particularly in proliferating cells. For this reason, ribosome synthesis is tightly regulated to maximize functional efficiency of cells [13–16].

Nucleoli disassemble as cells enter mitosis and then reform as cells enter the G1 phase. rDNA regions do not undergo typical chromosome condensation as cells enter mitosis. Instead, these regions remain loaded with upstream binding factor (UBF) and basal transcription machinery such as selective factor 1 (SL1), despite the lack of rDNA transcription [17–21]. This distinct chromosome feature results in a unique mitotic morphology attributed to rDNA containing regions termed secondary constrictions [22]. The transcription of rDNA can be activated during mitosis when cdc2-cyclin B kinase is inhibited [23], demonstrating the readiness of rDNA chromatin for transcription and further underscoring the importance of ribosome synthesis in cycling cells. During G1, rDNA spreads out at the p-arms of the five acrocentric chromosome pairs (chromosomes 13, 14, 15, 21, and 22) [24] and comes together to form nucleoli [25].

In many ways, nucleolar structure reflects the process of ribosome synthesis [7]. When examined by electron microscopy (EM) [26], nucleoli are recognized to contain three distinctive structures: fibrillar centers (FCs) believed to contain rDNA, dense fibrillar components (DFCs) believed to be the sites of transcription and processing of newly synthesized pre-rRNA, and granular components (GCs) where the ribosomal RNA and proteins form pre-ribosomes. Thus, the three nucleolar components represent the successive steps of ribosome biogenesis from rDNA to pre-ribosomal particles. Recently, a new compartment called the periphery of the dense fibrillar component was identified to contain proteins that ensure the anchoring of the 3′ end of rRNA for the accurate processing and removal of 3′ ETS [27]. When nucleoli form de novo after mitosis, the assembly of typical nucleoli with the characteristic EM tripartite structures (FC, DFC, and GC) is dependent on Pol I transcription [28–34]. Early studies demonstrated that various drugs with Pol I transcription inhibitory effects induced the segregation of nucleoli, a phenotype where the fibrillar and granular structures part from each other to form fibrillar caps around remanent nucleoli [32,35–37]. Nucleolar caps contain several factors that primarily pertain to Pol I transcription and pre-rRNA processing [38]. However, most of the tested Pol I inhibitory drugs were also multi-functional and genotoxic, which calls into question the specificity of these findings [36]. More recently, experiments using siRNA knockdown or AID-induced Pol I subunit degradation have further confirmed that the loss of Pol I transcription factors within 3 h is sufficient to induce the segregation of nucleoli and form nucleolar caps [33,34]. In comparison, interference of pre-rRNA processing does not appear to change nucleolar structure in terms of the tripartite organization as defined by EM [33], but the reduction in specific processome factors affects nucleolar number and size [14,39]. As ribosome biogenesis requires proper functioning of all three RNA polymerases, there is evidence that changes in RNA polymerase II (Pol II) activity could also influence rDNA transcription and alter nucleolar structure. Cells that were treated with the Pol II-specific inhibitor, a-amanitin, showed a substantial disruption of nucleolar structure as first reported in the 1970s [40]. More recently, non-coding RNA transcribed from Pol II, including Alu elements and others, has been shown to play roles in regulating Pol I transcription, pre-rRNA processing, and nucleolar structures [41].

In addition to being the site of ribosome synthesis, nucleoli are multi-functional organelles involved in cellular processes beyond ribosome synthesis [3,4,42]. Cellular activities reported to be associated with nucleoli include E3 ubiquitin-protein ligase MDM2-mediated regulation of p53 [43,44], stress response [45,46], macromolecular trafficking, assembly of various ribonucleoprotein particles, microRNA metabolism [47], genome organization, and others [3,48–54]. Whether these processes only use nucleoli as a host to facilitate their independent functioning or relate to ribosome synthesis directly is not clear. For example, MDM2 is primarily located in the nucleoplasm, and its regulation of p53 function is directly related to ribosome synthesis. Nucleolar stress due to a ribosome synthesis defect activates specific ribosomal proteins (primarily RPL11 and RPL5) to mediate the relocation of MDM2 to the nucleoli, release p53 from the negative control of MDM2, and initiate p53-mediated responses, often leading to apoptosis [44,55]. However, MDM2 could also be regulated by events other than ribosome synthesis. Recent studies reveal that MDM2 plays complex roles in cellular functions independent of p53, particularly during carcinogenesis. MDM2 may be regulated by factors such as MYC, NFAT1, IRF8, and others, and can influence various substrates in p53-deficient cells, exerting both oncogenic and tumor-suppressive effects depending on the context [56]. In another example, signal recognition particles (SRPs) are assembled in nucleoli, but it is not clear how SRP assembly in nucleoli relates to ribosome synthesis [57]. Furthermore, the attraction of heterochromatin to the surface of nucleoli has yet to be directly connected with ribosome synthesis. Initial studies in yeast have shown that nucleoli negatively influence the activities of the genome and silence Pol II transcription [58]. Over the past ten years, a substantial portion of mammalian genomes has been found associated with nucleoli and has been termed nucleolar-associated domains (NADs), which contain predominantly heterochromatin regions of each chromosome [53,54]. More recent studies show that the composition of NADs changes in cells at various stages of development [51,59]. How these changes [49–51,60] relate to ribosome synthesis at different cellular states remains to be explored. Interestingly, a substantial number of NADs overlap with domains associated with nuclear lamina (LAD) [53,61]. Live cell studies have demonstrated that chromosome domains at the nuclear or nucleolar periphery can interchange within the same cell cycle or more prominently across the cell cycle after mitosis [62]. In this study, perinucleolar or nuclear lamina-associated heterochromatin foci (GFP Dendra2-H3) were photoconverted with the smallest area laser exposure, and photoconverted signals were monitored through time-lapse confocal microscopy. Movement of the signals between nucleoli and nuclear periphery was observed, and these movements were particularly prevalent in nucleolar-nuclear lamina position changes across mitosis. There are many fewer cases where this movement takes place in interphase cells. Interestingly, Lamin A is not only localized to the nuclear periphery but can also be detected in the nucleoplasm and nucleolar periphery, which can help explain the partial overlap of NADs and LADs [63,64]. These findings underscore the dynamic nature of the 4D (four-dimensional) nucleome and the importance of nucleoli in genome organization.

## Regulation of ribosome synthesis in the nucleolus

rDNA transcription is considered the rate-limiting step in the regulation of ribosome synthesis. The epigenetic state of rDNA chromatin is heavily modulated by many intra- and extra-cellular regulatory mechanisms. Cell signaling pathways related to proliferation can either positively or negatively affect the transcription state of rDNA [65–69]. Unlike coding genes, which are typically in either active or inactive states, rDNA can be in three different states: highly methylated and condensed rDNA that are not likely to be activated, rDNA that are primed to be activated, and actively transcribed rDNA [70,71]. The super-redundant nature of rDNA presents distinct flexibility in the number of copies of rDNA that can be activated at any given stage or time in cells. Most recently, the copy number variation of rDNA has been found to be associated with body mass index in adult humans [24,68]. In addition, while studies from plants to *Xenopus* have described heritable dormancy of lineage-specific rDNA copies, this aspect is poorly understood in human cells [72]. The epigenetic state of rDNA chromatin dictates the transcriptional activity of the rDNA, limiting the rate of ribosome biogenesis [71,73,74].

The transcription and processing of pre-rRNA in the nucleolus is a co-ordinated process [75–77]. Efficient transcription involves a set of known factors that are also part of pre-rRNA processing machinery. Some of the essential processing factors, including a subset of t-UTPs that are components of pre-rRNA processing complexes, are also involved in Pol I transcription activation by acetylating UBF [75,78]. In yeast, the loss of Pol I transcription inhibits pre-rRNA processing. The complex RNA and protein composition involved in the multiple processing and assembly steps required for ribosome subunit formation mandates close regulation. The co-ordination of ribosome subunit production ensures that an equivalent number of small and large ribosome subunits are made, guaranteeing the successful assembly of these complex protein-making machines [11].

## Nucleolar structure and function in cancer vs. normal cells

Changes in nucleoli are one of the key characteristics of cancer cells that distinguish them from normal cells. Alterations in the size, number, or shape of cancer cell nucleoli in tissue samples have been reported more than a hundred years ago [7,79]. In fact, such alterations are clinically significant in several tumor types [79–81]. The specific changes in nucleoli most likely correspond to the dependence of cancer cells on ribosome synthesis for perpetual proliferation and the unique, cancer-specific regulation of this process [8,82,83]. Most terminally differentiated cells in tissues are post-mitotic and no longer go through the cell cycle. The amount of ribosome synthesis is typically significantly reduced in normal differentiated cells compared with cycling tumor cells. Mitogenic factors, including a number of oncogenes, such as MYC and RAS, serve to activate rDNA through cell signaling pathways, leading to desired epigenetic modifications and the activation of Pol I transcription [66,71,73,84,85]. In contrast, tumor suppressors such as Rb negatively regulate transcription activation [86–88]. The balance of signaling events based on the need for cell proliferative or metabolic states maintains the optimal ribosome synthesis regulation for a given cellular population. In cancer cells, such a balance tips toward an increase in ribosome biogenesis to meet the needs for cancer-specific metabolic activities [66]. In addition to enhanced ribosome output, recent findings suggest that there exists a population of cancer-specific ribosomes, oncoribosomes, produced preferentially in cancer cells that facilitate the expression of proteins that play key roles in cancer metastasis or drug resistance [89,90]. Increasing evidence indicates that not all ribosomes are equal. There is substantial variability in ribosomes at the rDNA level, in which ribosome variants could preferentially translate mRNAs [91]. Thus, ribosome biogenesis in cancer cells could produce ribosomes that not only serve to sustain proliferation but also play a key role in producing specific proteins important for the malignant behaviors of cancer cells [83,90].

Although much work has been done on the regulation of rDNA transcription activation and its co-ordination with pre-rRNA processing [75], less is known regarding how the subsequent steps of ribosome biogenesis and translation are co-ordinated. With the increased output of pre-rRNA, one could expect the corresponding enhancement of the machinery and output for the downstream steps. These steps include the assembly of pre-ribosomal particles with ribosomal proteins that are controlled by Pol II transcription, 5S rRNA transcribed by polymerase III (Pol III), transport of the pre-ribosomal subunits, final maturation, and translational engagement. How are the many steps of ribosome synthesis co-ordinated? One can imagine at least two possibilities. First, along with enhanced Pol I transcription, Pol II and Pol III are also activated through possibly the same mitogenic regulatory mechanism [71]. Thus, cancer-specific signaling could simultaneously regulate all steps of ribosome synthesis through modulating the expression of factors involved in ribosome synthesis. Secondly, it is also possible that the enhanced Pol I transcription alone could trigger the corresponding changes in the expression of factors required for ribosome making while simultaneously playing a role in carcinogenesis. Furthermore, there are also instances where factors involved in ribosome biogenesis can also be oncogenic. For example, pre-rRNA processing factor DEF, often overexpressed in cancers, plays a key role in the MYCN-driven neuroblastoma in a transgenic zebrafish model [92]. However, some proteins involved in ribosome synthesis are multifunctional; beyond assembling ribosomes, they also take part in other cellular processes that possibly operate independently of ribosome production in cancer cells. For example, NPM1, a protein enriched in the nucleolus, is involved in the assembly of ribosomal particles and also plays a separate role in DNA damage repair [93]. Mutants of NPM1 are oncogenic in several types of cancers and are under consideration for targeted drug development [94].

Other than making ribosomes, the nucleolus is a multi-functional organelle involved in the cell cycle, stress sensing, genome organization, assembly of SRPs, microRNA metabolism, and 4D organization of the genome and nucleus. It is highly probable that changes in nucleolar number, size, or structure could also influence the ways these functions contribute to the development of cancerous cells. Given that many microRNAs can act as either tumor promoters or suppressors, the regulatory impact of nucleoli on microRNAs can influence tumorigenesis [47,95]. In addition, nucleoli are stress-sensing organelles and have been reported to play a role in chemoresistance against cancer drug treatment [89,96]. Overall, nucleoli could contribute to carcinogenesis through multiple functions, including ribosome and/or oncoribosome synthesis, genome organization, stress sensing, metabolic response, small RNA regulation, and macromolecular trafficking controls.

## The pros and cons of cancer therapeutics that affect ribosome synthesis and nucleoli

Some of the classical anticancer chemotherapeutic drugs that have long been used clinically demonstrate inhibitory activities in ribosome synthesis and/or functioning [36]. These compounds include alkylating agents (cisplatin and oxaliplatin), intercalating agents (doxorubicin, mitoxantrone, actinomycin D, and mitomycin C), topoisomerase inhibitors (etoposide and camptothecin), and antimetabolics (methotrexate). In addition, some drugs affect early or late pre-rRNA processing events, such as 5-fluorouracil and MG-132, while others inhibit translation, such as cycloheximide and homoharringtonine [36]. Pol I transcription disruption coincides with nucleolar segregation, often exhibited by fibrillar components forming caps around the remnants of nucleoli [32]. Most prominently seen in drug-treated cells, the nucleolar cap can contain not only nucleolar fibrillar proteins (such as TBP, TAFII, and UBF), Pol I, fibrillarin, and Nop140 but also proteins that are part of other nuclear bodies, including Cajal bodies, PML bodies, speckles, and perispeckles [38], suggesting a functional association of nucleoli and other nuclear domains. This idea is further supported by a recent report where the siRNA knockdown of endogenous Pol I induced the capping of nucleoli, as well as a host of changes of other nuclear bodies and genome organization [33]. When pre-rRNA processing is inhibited, the tripartite nucleolar structures don’t always change [36], but the number and size can be altered. Studies using siRNA libraries to screen for factors required to maintain the number of nucleoli found that several processing factors are among those with critical roles in regulating nucleolar number [14,39]. When protein translation is inhibited by either cycloheximide or homoharringtonine, significant alteration of nucleolar structure is not observed [36].

Many clinical chemotherapeutic drugs have been used to successfully prevent cancerous cell proliferation. Even though ribosome synthesis often was not the intended target for these drugs, they have similar ribosome synthesis inhibitory effects [36]. The use of these drugs has improved patient survival rates, and these compounds are still clinically available for those who have limited options. However, these drugs often have multifaceted effects on both cancer and normal cells. Compounding with the cytotoxic principle, treatment with these drugs often results in severe adverse effects on patients.

## Pol I inhibitors CX-5461 and BMH21

Over the past 20 years, specific Pol I inhibitors have been developed to decrease the general cytotoxicity of existing drugs [97]. Cell biochemical-based screening assays and medicinal optimizations produced the Pol I inhibitor CX-5461 [98–102]. This compound was initially shown to be a competitive inhibitor, preventing the preinitiation complex from binding to rDNA [98] and reducing rDNA transcription in patients in a Phase I clinical trial [103]. CX-5461 has been demonstrated to induce DNA damage responses [104] and tumor-specific p53 activation [105]. More recently, CX-5461 has been found to be a G-quadruplex stabilizer [106,107] and a TOP2B inhibitor [108], both of which could also be part of the mechanisms for Pol I transcription inhibition. It would not be surprising if additional mechanisms of action could surface in the future. CX-5461 has passed a Phase I clinical trial, showing some efficacy against solid tumor growth [106]. Even though this compound was derived from Pol I transcription inhibition screens, the mechanisms of action could extend beyond interferences with ribosome synthesis [106–108].

Another Pol I inhibitor, BMH21, was developed from a cell-based screen for small molecules that activate p53 [109]. Initially, BMH21 was not considered a DNA damage agent, and the treatment induces a rapid degradation of Pol I large subunit RPA194 [110,111]. The inhibition of rDNA transcription by BMH21 is conserved from yeast to mammalian cells [111,112], providing a range of model systems to determine the mechanisms of Pol I transcription inhibition by this drug. A more recent report showed that BMH21 reduces initiation, promoter escape, elongation, and Pol I occupancy on the rDNA. The retardation of Pol I elongation is mediated by extending polymerase pausing preferentially at the region upstream of GC-rich sequences [111,113]. The preinitiation factors UBF, RRN3, and SL1 are required for the degradation of Pol I large subunit [111]. BMH21 is currently in a pre-clinical development stage.

## Development of metarrestin

Metarrestin (ML246), another Pol I transcription inhibitor, is an effective metastasis inhibitor [114]. Although it inhibits Pol I loading to rDNA, the development of metarrestin was not derived from targeting rDNA transcription. Instead, it was discovered through an alternative and holistic approach based on the hypothesis that cancer cells may share unique nuclear structural characteristics that are absent in normal cells. To identify such characteristics, we immunized mice with HeLa nuclei and established a hybridoma library from the immunized mouse plasma cells (our unpublished data). The culture supernatant of each clone in the library was screened by immunofluorescence to search for antibodies that labelled specific nuclear structures present in HeLa cells but absent in NIH3T3 cells (a proliferating normal mouse fibroblast). The initial screen identified an antibody from the SH54 hybridoma cell clone that immunolabeled the perinucleolar compartment (PNC) [115]. Subsequent evaluation of many cancer cell lines derived from solid tissue origins and normal cells, including embryonic stem (ES) cells, demonstrated the unique presence of PNCs in cancer cells and absence from normal cells, including ES cells [116]. Further investigations using patient tumor banks with follow-up information showed that PNC prevalence (percentage of cells with at least one PNC) is positively correlated with disease progression and negatively associated with patient outcomes in several aggressive cancers [117,118]. The association of PNC prevalence with metastatic potential in both cultured cells and tumor tissues suggests that PNC prevalence can be used as a surrogate marker for cancer metastasis [116].

Given that cancer metastasis is complex and heterogeneous, and that the factors necessary and sufficient for this process remain unknown, a complex cellular structure should reflect the cancer cell’s unique traits more comprehensively than any single gene or gene product. Therefore, we used PNC prevalence as a surrogate marker for cancer metastasis during drug development. We established a single-step, high-content, cell-based assay to screen a library of 140,000 diverse compounds for those that reduced PNC prevalence by 50% based on the idea that PNC-containing cells are metastatic-capable cells [119]. The reduction in cells containing PNCs would signify either selective killing of PNC-positive cells or disruption of cancer-specific behaviors as reflected in the loss of PNC structure maintenance.

The use of such a phenotypic marker for selective anticancer metastasis drug development has several advantages. Small-molecule drugs are generally promiscuous in that each often binds many cellular molecules with varying affinities in addition to their specific target [120–122]. Screening using a phenotypic marker, particularly for a complex disease such as cancer, could result in hits that bind multiple functionally relevant targets, which may be required synergistically to support a cancer metastasis phenotype. This idea is highly probable, as no single gene or gene product has so far been found to be necessary and sufficient for cancer metastasis despite intense research over the past few decades, underscoring the complexity of the phenotype likely to be contributed by multiple factors and pathways regulated in cancer-specific ways. As a proof of principle, the primary screen against PNCs, subsequent extensive secondary and tertiary screens, and medicinal chemistry development yielded metarrestin [114,123–125], a potent PNC and selective metastasis inhibitor *in vitro* and *in vivo* that is currently in clinical trial for pancreatic cancer treatment. Metarrestin selectively inhibits metastatic cancer growth with negligible impact on primary tumors in metastatic pancreatic and prostate cancer xenograft models. The treatment of metarrestin in tumor-bearing mice provided 100% survival benefit when treatment commenced prior to macro-metastasis development and a significantly enhanced survival rate when treatment was initiated after macro-metastasis developed in the pancreatic xenograft model. More importantly, the cause of death for the mice that did not survive in the post-metastasis treatment was primarily from the growth of the primary tumors. Post-mortem necrotic analyses showed that the metarrestin-treated group had minimal metastatic tumor burden in livers and lungs as compared with the vehicle-treated group, where the organs were completely displaced by metastatic tumor masses [114]. The selective inhibition against metastasis by metarrestin provides supporting evidence that PNCs could serve as a viable biomarker for anticancer metastasis drug development.

Although the precise mechanisms of action for metarrestin remain to be understood, the drug is a potent rDNA transcription inhibitor, which occurs through the reduction in Pol I polymerase occupancy on rDNA without a significant impact on UBF binding [114]. Metarrestin affects Pol I transcription in tumor cells more effectively than in normal cells at the same treatment concentration. However, the mechanism by which metarrestin reduces Pol I occupancy on rDNA remains a subject of current research. The disruption of the PNC closely corresponds to the inhibition of Pol I transcription, which induces nucleolar segregation [114]. As the inhibition of Pol I with siRNA could also reduce PNC prevalence, it is highly suggestive that PNC disruption by metarrestin could be mediated by Pol I transcription inhibition and nucleolar segregation [114]. Unlike other Pol I inhibitors, metarrestin treatment does not induce DNA damage repair responses or the activation of p53. Cells treated with metarrestin generally don’t undergo apoptosis [114]. In addition to Pol I inhibition, metarrestin also activates autophagy [126], and treatment with the drug is protective for memory loss in a neurodegenerative mouse model [127]. As metarrestin was derived from a phenotypic marker screen, it could target multiple functionally important cellular factors or pathways, including ribosome synthesis. Studies are underway to identify the critical players that mediate the antimetastatic efficacy of metarrestin.

While PNCs are associated with metastatic behavior [114,119,124,125], their molecular composition and function in metastatic cancer cells remain poorly understood. The PNC contains RNA transcribed by Pol III (RMRP, RPPH1, 7SL, HY1, 3, and 5) [128–130] and Pol I (PNCTR) [131], along with RNA-binding proteins such as polypyrimidine tract-binding protein (PTBP1) and CEF1 [115,130,132]. PNCs are highly enriched with newly synthesized RNA [133] and are associated with DNA domains [134], suggesting possible roles in transcription and RNA metabolism. Although PNCs are physically associated with nucleoli, many nucleolar proteins and RNAs are not enriched in PNCs. These factors include Pol I transcription factors (UBF and RPA194), pre-rRNA processing factors (fibrillarin, Nopp140), and rRNA (18S and 28S RNA) (our unpublished data). The only nucleolar protein found enriched in the PNCs thus far is nucleolin (our unpublished data). It remains unclear why PNCs are physically associated with nucleoli and how they are functionally related. It is noted that because PNCTR is transcribed by Pol I [131], PNCTR could be concentrated in PNC post-transcriptionally. Studies are underway to resolve the molecular composition and interactions in PNCs to address the functional association of PNCs with nucleoli, ribosome synthesis, and cancer metastasis. It remains clear, however, that PNCs are dependent on Pol I transcription. In addition to metarrestin, nearly all Pol I inhibitors eliminate PNCs, including CX-5461, BMH21, cisplatin, actinomycin D, and others (our unpublished data). These findings would suggest that PNC maintenance is highly dependent on Pol I transcription. However, whether Pol I transcription or nucleolar function can be influenced by PNCs, particularly in the context of cancer, is an interesting question to address.

## Summary

Ribosome synthesis and nucleoli are significantly altered in cancer cells compared with normal cells. However, it is not clear whether ribosome synthesis is among the initiators of carcinogenesis or an essential requirement for perpetual cell proliferation during tumor growth. There is evidence that factors in ribosome synthesis or specific ribosome variants play roles in promoting carcinogenesis. While there are justifications to encourage the strategy of targeting nucleoli and ribosome biogenesis in anticancer drug development, it is difficult not to entertain the possibility that enhanced ribosome synthesis during carcinogenesis is the result of a pathological regulatory signaling package that pushes the cell transformation toward cancer. It is also crucial to recognize that ribosome synthesis is one of the many nucleolar functions, and that nucleolar structures may not only reflect ribosome synthesis but also have a significant role in other nuclear functions globally. Thus, the inhibition of ribosome synthesis could affect not only ribosome assembly but also the cellular processes required for cancer growth and metastasis. Given that drugs which inhibit rDNA transcription or other steps of ribosome synthesis have so far proven to be clinically effective against various solid tissue-derived cancers, there is no doubt that ribosomal synthesis is a crucial process in maintaining cancer growth. However, given that this process is also important for normal cycling cells, these drugs typically have substantial adverse effects in patients due to the lack of discrimination against tumor and normal cycling cells.

**Figure 1:**
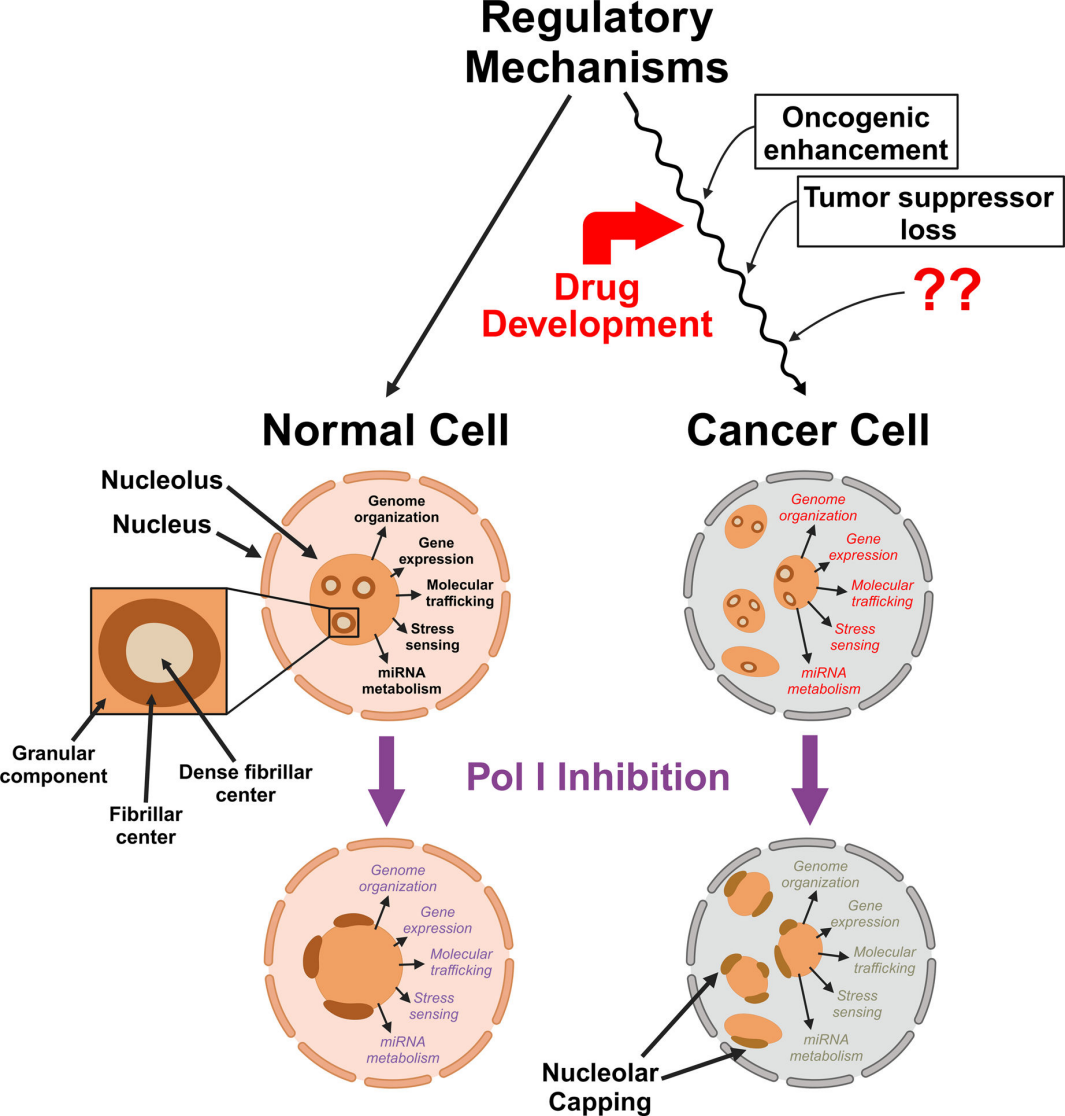
The structure and function of nucleoli differ between cancerous and normal cells. Cancer cells generally have more nucleoli and larger nucleolar areas when compared with normal cells. In addition to ribosome synthesis, nucleoli play a role in the nucleoplasmic regulation of genome organization, gene expression, macromolecular trafficking, stress sensing, and miRNA metabolism, all processes that are also altered in cancerous cells to facilitate increased cellular proliferation and malignant behavior. Ideally, chemotherapeutic drug development should aim to target cancer-specific regulatory mechanisms that contribute to the enhanced cancer-specific ribosome synthesis to increase the selectivity of drugs against cancer cells over normal cells. Inhibition of RNA Pol I in both normal and cancerous cells results in damage to the nucleolar structure and the development of nucleolar caps. Disruption of nucleolar structure from RNA Pol I inhibition affects the ability of nucleoli to properly perform their multifunctional roles in both normal and cancerous cells. Created in BioRender [135].

Ideally, anticancer drug development against ribosome synthesis should focus on cancer-specific regulation of ribosome synthesis (Figure 1), so that the successful drugs inhibit ribosome biogenesis preferentially in cancer cells over normal cells. Such selectivity would reduce adverse effects of the treatment for cancer patients. The development of metarrestin represents an example of such an approach. Metarrestin was identified through a screen against a cytological marker indicative of the metastatic phenotype. This novel process offers an opportunity to identify effective chemotypes that could potentially target multiple functionally relevant cellular processes required to synergistically promote metastasis. While metarrestin blocks Pol I loading onto the rDNA, it does not appear to bind Pol I directly, suggesting interference at the upstream elements of ribosome synthesis in cancer cells. Studies are underway to investigate the mechanism by which metarrestin inhibits ribosome biogenesis. With recent advances in AI and bioinformatics, there are now tools to extrapolate and identify key players involved in cancer-specific ribosome synthesis and associated regulatory mechanisms, thus providing the fundamental information essential for the development of selective anticancer and antimetastatic compounds.

**Perspectives**

- Cancer growth and metastasis remain an unsolved clinical problem, as metastasis is the leading cause of death for patients with solid tissue-derived tumors. Despite over half a century of intense research efforts, the key mechanisms necessary and sufficient for metastasis are still unknown. Ribosome biogenesis and nucleoli have been shown to be altered and enhanced in cancer cells, leading to the hypothesis that they may play an important role in carcinogenesis. Drugs that disrupt ribosomal DNA transcription have shown efficacy against cancer growth and are the standard of care for cancer patients with limited options. However, the indiscriminate inhibition of ribosome synthesis in both cancer and normal cells can result in severe adverse effects.
- To reduce the nonspecific toxic effects of chemotherapeutics, anticancer drugs that disrupt ribosome synthesis, specifically polymerase I transcription, are currently being developed. The three leading compounds (CX-5461, BMH21, and metarrestin) have been shown to interfere with ribosome synthesis, two of which are currently in clinical trials.
- Ideally, anticancer drug development should consider targeting tumor-specific regulatory elements that enable the enhancement of ribosome synthesis and nucleolar structure, two of the key players in cancer development and metastasis. Contrary to indiscriminate cancer treatments, perturbing these cancer-specific mechanisms could substantially mitigate the adverse effects of anticancer treatment on normal cells.

## Abbreviations

4D: four-dimensional
DFCs: dense fibrillar components
EM: electron microscopy
ES: embryonic stem
FCs: fibrillar centers
GCs: granular components
NADs: nucleolar associated domains
PNC: perinucleolar compartment
Pol I: polymerase I
Pol II: polymerase II
Pol III: polymerase III
SL1: selective factor 1
SRPs: signal recognition particles
UBF: upstream binding factor
rDNA: ribosomal DNA
