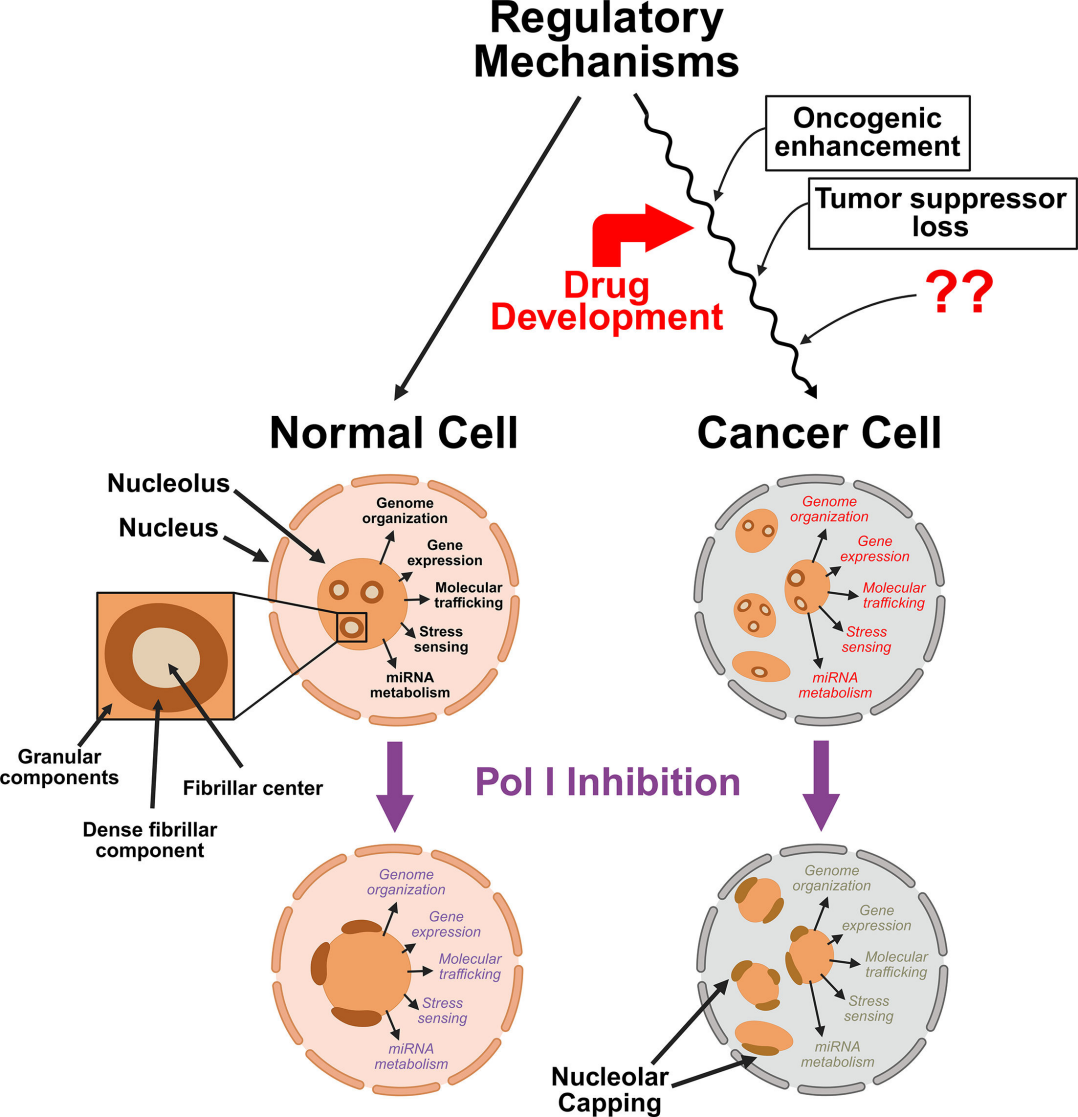# Correction: Anticancer drug development against ribosome synthesis and the nucleolus

**DOI:** 10.1042/BST20253011_COR

**Published:** 2025-10-13

**Authors:** Andrew Loiacono, Sui Huang

**Affiliations:** Department of Cell and Developmental Biology, Northwestern University Feinberg School of Medicine, Chicago, U.S.A.

The authors of the original article “Anticancer drug development against ribosome synthesis and the nucleolus” 10.1042/BST20253011 would like to correct Figure 1.

The ‘Fibrillar Center’ was originally mislabelled as the ‘Dense Fibrillar Center’, and the ‘Dense Fibrillar Component’ was originally mislabelled as the ‘Fibrillar Center’. These labels have been changed in the corrected figure.

The requested correction has been assessed and agreed by the Editorial Board. The authors declare that these corrections do not change the results or conclusions of their paper. The corrected version of Figure 1 is presented here:

**Figure 1:**